# Influence of family planning and immunization services integration on contraceptive use and family planning information and knowledge among clients: A cross-sectional analysis in urban Nigeria

**DOI:** 10.3389/fgwh.2022.859832

**Published:** 2022-09-20

**Authors:** Kate L. Sheahan, Ilene Speizer, Siân Curtis, Morris Weinberger, John Paul, Antonia V. Bennett

**Affiliations:** ^1^Department of Health Policy and Management, Gillings School of Global Public Health, University of North Carolina at Chapel Hill, Chapel Hill, NC, United States; ^2^Department of Maternal and Child Health, Gillings School of Global Public Health, University of North Carolina at Chapel Hill, Chapel Hill, NC, United States

**Keywords:** family planning, child immunization, integration, Nigeria, Sub-Saharan Africa

## Abstract

Reproductive autonomy necessitates that women have access to high quality family planning information and services. Additionally, closely spaced pregnancies increase maternal and infant morbidity and mortality. Although integrating family planning into child immunization services may increase access to information and services and postpartum contraceptive use, evidence on how integration affects service delivery and health outcomes is scarce. One limitation of previous studies is the use of binary integration measures. To address this limitation, this study applied Provider and Facility Integration Index scores to estimate associations between integration and contraceptive use, receipt of family planning information, and knowledge of family planning services availability. This study leveraged pooled cross-sectional health facility client exit interview data collected from 2,535 women in Nigeria. Provider and Facility Integration Index scores were calculated (0–10, 0 = low, 10 = high) for each facility (*N* = 94). The Provider Integration Index score measures provider skills and practices that support integrated service delivery; the Facility Integration Index score measures facility norms that support integrated service delivery. Logistic regression models identified associations between Provider and Facility Integration Index scores and (a) contraceptive use among postpartum women, (b) receipt of family planning information during immunization visits, and (c) correct identification of family planning service availability. Overall, 46% of women were using any method of contraception, 51% received family planning information during the immunization appointment, and 83% correctly identified family planning service availability at the facility. Mean Provider and Facility Integration Index scores were 6.46 (SD = 0.21) and 7.27 (SD = 0.18), respectively. Provider and Facility Integration Index scores were not significantly associated with postpartum contraceptive use. Facility Integration Index scores were negatively associated with receipt of family planning information. Provider Integration Index scores were positively associated with correct identification of family planning service availability. Our results challenge the position that integration provides a clear path to improved outcomes. The presence of facility and provider attributes that support integration may not result in the delivery of integrated care.

## Introduction

The Government of Nigeria has committed to improving health outcomes for women and infants but has struggled to reduce maternal and infant mortality rates (1). In 2017, Nigeria's maternal mortality rate (MMR) was the fourth highest in the world at 917/100,000 (2). In 2019, its infant mortality rate (IMR) was 76/1,000 live births, the third highest in the world (3). Nigeria aims to promote healthier outcomes among women and children by increasing access to and use of contraception (4). To support this aim, all people need to have the fundamental right to decide the number, timing, and spacing of their children and have access to high quality contraceptive information and services (5). Among other priorities, the Nigerian Government has recognized the importance of satisfying unmet need for contraception among postpartum women (6, 7). In addition to promoting reproductive rights and autonomy, access to family planning methods in the postpartum period is important because pregnancies that are spaced too closely together are associated with increased risk of an array of adverse outcomes, including low birth weight, fetal death, and neonatal death (8–10). Research in sub-Saharan Africa, and specifically in Nigeria, shows that few women who have given birth within the last year wish to have another birth within 2 years and that unmet need for contraception is as high as 59% among women who have given birth within the last year (11–13).

The Nigeria Ministry of Health (MoH) promotes integration of family planning into child immunization services because of its potential to increase access to contraception among postpartum women and thus promote improved maternal and infant health outcomes (7). The Minimum Standards for Primary Healthcare (MSPH) in Nigeria identify both family planning and immunization services as minimum components of primary healthcare and require provision of these services at all public primary healthcare facilities while advocating that privately owned facilities align with these standards (14). The Nigerian Urban Reproductive Health Initiative (NURHI) bolstered the MoH's integration strategy within the facilities it supported by incorporating the following family planning approaches into immunization services: (a) provision of information, education, and counseling materials on all methods; (b) group counseling; and (c) referral of prospective clients to the family planning clinic (15).

Integration leverages the routine immunization schedule recommended at birth, 6 weeks, 8 weeks and at several intervals until ~12 months, by repeatedly offering family planning information and services to postpartum women at these visits (16). While integration seems promising, evidence about its effects is scarce and conflicting. Some research reports that integrated services may be responsive to women's preferences and that integration offers repeated opportunities to provide information to women and may increase contraceptive prevalence without detriment to immunization rates (17–22). However, other studies show no significant increase in family planning use when women receive family planning information and referrals during immunization visits (23–25). Additionally, integration approaches vary, and the effects may be heavily dependent on context (26). It is critical to tailor interventions to the context and implement approaches that do not compromise either family planning or immunization services. For example, integrating these two services may erode community trust. This is particularly relevant in northern Nigeria, where communities have boycotted immunizations because of widespread belief that immunizations have been infused with anti-fertility drugs (27). To justify the service delivery reorganization that family planning and immunization integration requires, it is critical to better understand the relationship between integration and important health and service delivery outcomes, including postpartum contraceptive use and service quality (26, 28–32).

Information provision is an essential component of quality family planning service delivery that enables clients to confidently choose and competently use a method of their choosing, and also to understand the support and services that they can expect from their health facility (33). Sharing of information about family planning options may also increase knowledge and enable an exchange about women's fertility and method preferences, which promotes reproductive rights and autonomy, regardless of whether a woman opts to utilize a method or not (34). A robust body of research establishes the connection between quality of family planning services and use of contraception (35–38). However, little evidence exists about the relationship between integration and information provision. Some research highlights the challenges associated with providing individualized family planning information during immunization appointments and questions the feasibility of doing so (24). Similarly, a review by Dudley et al. (39) found that integrated family planning and maternal and child health services may lead to decreased patient knowledge about family planning.

While this paper focuses on the integration of family planning into immunization services, the potential benefits and challenges of service integration are pertinent across health areas and systems (40). On one hand, integration may be acceptable to providers and clients and can result in a range of benefits, including cost efficiency, reduced stigma, and increased access to information and care (25, 41–43). On the other hand, ambiguous guidelines, inadequate training, and heavy workloads shouldered by providers challenge integration (44, 45). Existing vertical information, logistic, and funding systems further hamper efforts to scale integrated service delivery (46, 47). Considering both the potential and the challenge of integrating family planning into immunization services, more evidence is needed to identify whether and how this approach can deliver intended benefits to individuals and health systems.

While most studies measure integration as a binary (generally defined as whether a facility implemented strategies to increase integration), the degree of integration attained within facilities is actually multi-dimensional and varies over time and across facilities (48, 49). No known studies have used continuous or ordinal measures of family planning and child immunization integration to estimate associations between the degree of integration of service delivery and behavioral outcomes. This study addressed that gap by identifying associations between Provider and Facility Integration Index scores and (a) contraceptive use among postpartum women, (b) receipt of family planning information during immunization visits, and (c) correct identification of family planning service availability at the facility the women attended to attain immunizations for their child.

The study protocol and all consent procedures and consent forms were approved by the Institutional Review Board (IRB) at the University of North Carolina at Chapel Hill and by the National Health Research Ethics Committee of Nigeria in Nigeria. This secondary data analysis was reviewed by the UNC IRB and deemed exempt.

## Methods

### Data and sample

Data were collected at two time points for the impact evaluation of the Nigerian Urban Reproductive Health Initiative (NURHI) (50). NURHI aimed to increase contraceptive prevalence, particularly among the urban poor, and supported family planning service delivery in all facilities in our sample. Data for this study were collected from health facilities in Abuja, Benin City, Ibadan, Ilorin, Kaduna and Zaria. The facilities were high-volume (each saw more than 1,000 antenatal care patients per year), publicly and privately owned, and included both primary and secondary level facilities.

We use pooled cross-sectional client exit interview data from 2011 (112 facilities) and 2014 (132 facilities). The facilities were the same in 2011 and 2014, with some new facilities added in 2014. The exit interviews collected information from women regarding demographic and socioeconomic characteristics, family planning utilization, types of services sought, whether family planning information and services were offered to them during immunization visits, and satisfaction with services. All women aged 15–49 who attended a facility for maternal, newborn, and child health services as well as some related services (e.g., prevention of mother-to-child transmission of HIV) were eligible for interview. In our sample, we include women ages 15–49 who were attending facilities to attain child immunization services, were married or cohabiting with a male partner, not pregnant at the time of the interview and who had been pregnant at least once. We did not have data relating to timing of the last pregnancy. Therefore, given the recommended immunization schedule that a child completes around 12 months of age, we assumed that women attending the immunization visit had given birth within the last 2 years. Our analytic sample included 2,535 women: 1,393 women at 82 facilities in 2011 and 1,142 women at 94 facilities in 2014.

### Analytic method

We employed multivariate logistic regression models to investigate the associations between provider and facility integration and family planning outcomes (Equation 1):


$$\begin{array}{r} Y_{it}=\beta_{0}+\beta_{1}Int_{it}+\underset{k}{\sum }\beta_{k}X_{kit}+\varepsilon_{it} \end{array}$$

*Y*_*it*_ represented our three outcome variables of interest in individual *i* at time period *t*. The first outcome variable, use of any modern method to prevent pregnancy at the time of the immunization visit, was a binary variable that took the value of 1 if the woman indicated that she was using one or more of the following methods at the time of the immunization visit: implant, intrauterine device, injectable, oral contraceptive pills, emergency contraceptive pills, male and female condoms, the standard days method/Cycle Beads, or the lactational amenorrhea method (51). The second outcome was an indicator variable measuring whether the woman reported seeing or receiving any family planning information during their child's immunization visit. The third outcome variable measured whether the woman correctly identified family planning service availability at the facility. To construct this variable, we used responses from the client exit survey in which women were asked whether family planning services were available at that facility. We compared women's responses to facility survey responses, which capture family planning service availability. Matching responses were coded as correct identification of service availability. We used the client exit and provider interview data to describe family planning consultation content, utilization, and desire for family planning information.

Our independent variables of interest, *Int*_*it*_ included two measures of provider and facility family planning and child immunization services integration—the Provider Integration Index and Facility Integration indexes. The indexes were constructed using principal components analysis (PCA) with cross-sectional health facility (*N* = 400) and healthcare provider (*N* = 1,479) survey data that were collected in six urban areas of Nigeria for the NURHI impact evaluation. Development and application of these indexes has been detailed in previous papers (49, 52). The Provider Integration Index score (Cronbach's alpha = 0.88) reflects healthcare provider's skills and willingness to offer family planning information or services during a child immunization visit. It includes the following components: proportion of providers at facility that offer both child immunization and family planning services, proportion of providers at facility that routinely offers family planning information during immunization or child growth monitoring visits, proportion of providers at facility that do not request partner consent prior to woman's receipt of family planning services during child health visit, and facility provides both child immunization and family planning services. The Facility Integration Index (Cronbach's alpha = 0.76) score reflects operational management standards and procedures that support access to both child immunization and family planning services at the same facility, either at the same consultation or on the same day. It includes the following components: normal practice at this facility if client wants family planning information during child health visit, normal practice at this facility if client wants hormonal method of family planning during child health visit, and score of operational days when both immunization and family planning services are offered. Each index score ranges from zero (lowest level of integration) to 10 (highest level of integration). The integration indexes align with the guidance provided by Nigeria's Federal Ministry of Health and reflect critical attributes of integration identified in the literature (53–56). Additional information about the integration indexes is provided in the Appendix.

Because parity, education, wealth and desire for more children influence Nigerian women's use and knowledge of family planning, we included these as controls (*X*_*kit*_ in Equation 1) (57). As a sensitivity analysis we also conducted a stratified analysis to compare results between women who wanted and didn't want another child in the future. All standard errors ε_*it*_ were adjusted for clustering at the facility level. To test for non-linear association between the integration index scores and the outcome variables, we also ran models with the quadratic integration index terms. Inclusion of quadratic integration index variables to identify non-linear associations did not change the significance of results. We thus present results for models without these variables. Statistical significance was determined at alpha = 0.05. All analyses were conducted using Stata 13.1 (Stata Corp LP, College Station, Texas, USA).

## Results

Table 1 presents the characteristics of women in our sample. On average, women were 28 years old (SD = 5.2), with 2.45 living children. Three-quarters of the women had attended senior secondary school or higher. Overall, 72% of the women desired another child and 46% reported using any method to prevent pregnancy at the time of the immunization visit. Of those, 87% reported using at least one modern contraceptive method. Amongst women not doing anything to prevent pregnancy, the reasons included breastfeeding (34%), infrequent sex (18%), fear of side effects (12%), and desire to conceive (9.5%). The majority of women interviewed (89%) attended a public facility for the immunization visit. Just over half (56%) attended a primary care facility for the immunization visit. Half (51%) reported receiving family planning information during their immunization visit, and 83% correctly identified family planning service availability at the facility. The mean Provider and Facility Integration Index scores for the facilities attended by the women in our sample were 6.80 (SD = 2.1) and 7.3 (SD = 1.73), respectively.

**Table 1:** Characteristics of married/cohabitating postpartum women attending facility for child immunizations.

**Characteristic**	**% (*N* = 2,535)**
**Study outcomes**	
Doing anything to prevent pregnancy at time of immunization visit	46.31
Using a modern method of contraception at time of immunization visit (among those doing anything to prevent pregnancy)	87.00
Received family planning information during immunization visit	50.85
Correctly identified family planning service availability at facility	83.27
**Client characteristics**	
Age, mean (SD)	28.19 (5.21)
Would like to have another child	71.99
Muslim	37.59
Number of children, mean (SD)	2.45 (1.50)
Highest level of education	
None	3.20
Quranic only	2.13
Primary	10.85
Junior secondary	10.02
Senior secondary	44.50
Higher	29.31
Client household characteristics	
Toilet quality	
High quality	53.14
Latrine/poor quality	44.73
No toilet/latrine	2.13
Roof made with high quality material	89.47
Has improved water source	73.65
Has electricity	96.73
Has mobile phone	84.34
Has radio	90.49
Has improved cooking technology	34.48
Has TV	95.38
Has refrigerator	65.58
**Characteristics of facilities attended by clients**	
City	
Abuja	17.04
Benin	23.91
Ibadan	29.19
Ilorin	15.07
Kaduna	9.70
Zaria	5.09
Ownership	
Public	88.88
Private	11.12
Level	
Primary health center	55.98
Hospital	44.02
Provider Integration Index Score, mean (SD)	6.46 (0.21)
Physical Integration Index Score, mean (SD)	7.27 (0.18)

Table 2 presents the multivariate models for modern family planning use at the time of the immunization visit. We found no association between Provider (model 1) and Facility (model 2) Integration Index scores and client-reported use of a modern method of family planning at the time of the immunization visit (Provider Integration Index OR = 1.005, 95% CI = 0.900–1.122, *p* > 0.05; Facility Integration Index OR = 0.879, 95% CI = 0.772–1.001, *p* > 0.05). However, we note that the confidence interval of Facility Integration Index suggests a meaningful, if not statistically significant, association. Across both models, number of children is significantly associated with modern family planning use. Year is also significantly associated with modern family planning use in both models, indicating that modern family planning use increased in these facilities over the project period. Muslim religion is only significant in the Provider Integration Index model. Interestingly, age, education, wealth, and whether a woman wanted another child were not significant in either model. Across all models, results of the stratified analysis to compare results between women who wanted and didn't want another child showed no difference in integration scores and control variables between the groups.

**Table 2:** Association between provider integration index and facility integration index and use of modern family planning method at time of the immunization visit.

**Variable**	**Odds ratio (95% CI)**		**Odds ratio (95% CI)**
**Provider Integration Index Score (Model 1)**	1.005 (0.900–1.122)	**Facility Integration Index Score (Model 2)**	0.879 (0.772–1.001)
Age	0.987 (0.962–1.012)		0.986 (0.962–1.012)
**Education**
None	ref		ref
Quranic only	0.790 (0.225–2.767)		0.812 (0.235–2.800)
Primary	1.137 (0.472–2.738)		1.176 (0.502–2.754)
Junior secondary	1.249 (0.535–2.920)		1.299 (0.569–2.967)
Senior secondary	1.914 (0.799–4.585)		2.010 (0.862–4.689)
Higher	1.822 (0.728–4.561)		1.901 (0.785–4.607)
Want another child	1.032 (0.762–1.398)		1.049 (0.770–1.429)
Muslim	0.766^*^ (0.599–0.980)		0.795 (0.624–1.012)
Number of children	1.194^**^ (1.072–1.330)		1.197^**^ (1.073–1.335)
Wealth	1.005 (0.852–1.186)		0.996 (0.854–1.161)
Endline	5.669^***^ (3.502–9.174)		5.853^***^ (3.800–9.013)
**City**
Abuja	ref		ref
Benin	2.069 (0.935–4.577)		1.862 (0.959–3.614)
Ibadan	1.953 (0.968–3.940)		1.698^*^ (1.003–2.874)
Ilorin	0.882 (0.421–1.844)		0.814 (0.412–1.609)
Kaduna	0.453 (0.173–1.184)		0.448^*^ (0.202–0.993)
Zaria	1.005 (0.352–2.867)		0.848 (0.330–2.177)
Facility family planning client load	0.999 (0.993–1.004)		0.998 (0.994–1.003)
Public facility	1.570 (0.824–2.989)		1.719 (0.868–3.403)
Constant	0.0319^***^ (0.00749–0.136)		0.0788^**^ (0.0148–0.420)
Observations	2,530		2,530

*** p < 0.001,

** p < 0.01,

* p < 0.05,

wealth is measured with an index created using polychoric PCA based on household ownership of nine items.

Table 3 shows that Provider Integration Index (model 3) scores were not significantly associated with client reported receipt of family planning information during an integrated child immunization visit (OR = 0.973, CI = 0.842–1.124, *p* = 0.710) while higher Facility Integration Index (model 4) scores were associated with decreased odds of receipt of family planning information (OR = 0.835, 95% CI = 0.716 – 0.975, *p* < 0.05). Year was significantly associated with receipt of family planning information. Public facility was significant only in the Facility Integration Index model, while other controls were not significant in either model. Of the 49% of women in our sample who did not receive family planning information during the immunization visit, 41% reported that they would have been interested in receiving it. Those women primarily expressed interest in receiving information about injectables (49%) and oral contraceptive pills (23%). Of the remaining 59% not interested in receiving information, the most common reasons for lack of interest included current breastfeeding (28%), fear of side effects from contraception (19%), desire for another child (14%), or husband's disapproval (13%; data not shown). Additionally, providers reported discussing fewer family planning topics with clients during integrated consultations than during stand-alone family planning consultations (2.6 vs. 2.9, *p* < 0.001; data not shown).

**Table 3:** Association between provider and facility integration index scores and receipt of family planning information during immunization visit.

**Variable**	**OR (95% CI)**		**OR (95% CI)**
**Provider Integration Index Score (Model 3)**	0.973 (0.842–1.124)	**Facility Integration Index Score (Model 4)**	0.835^*^ (0.716–0.975)
Age	1.004 (0.982–1.028)		1.003 (0.980–1.027)
**Education**
None	Ref		Ref
Quranic only	1.145 (0.463–2.835)		1.195 (0.477–2.995)
Primary	0.873 (0.471–1.619)		0.934 (0.491–1.776)
Junior secondary	0.854 (0.463–1.575)		0.933 (0.490–1.775)
Senior secondary	1.135 (0.631–2.043)		1.261 (0.668–2.382)
Higher	0.975 (0.525–1.812)		1.055 (0.547–2.035)
Want another child	1.043 (0.777–1.400)		1.073 (0.794–1.449)
Muslim	1.096 (0.828–1.451)		1.189 (0.900–1.571)
Number of children	1.054 (0.953–1.166)		1.06 (0.957–1.174)
Wealth	1.040 (0.895–1.209)		1.046 (0.896–1.222)
Endline	5.939^***^ (3.256–10.83)		6.289^***^ (3.600–10.99)
**City**
Abuja	Ref		Ref
Benin	2.168 (0.820–5.734)		1.609 (0.577–4.485)
Ibadan	1.915 (0.821–4.464)		1.373 (0.750–2.513)
Ilorin	0.934 (0.405–2.156)		0.743 (0.345–1.600)
Kaduna	1.024 (0.433–2.419)		0.793 (0.372–1.691)
Zaria	1.082 (0.365–3.207)		0.727 (0.276–1.916)
Facility family planning client load	0.998 (0.990–1.006)		0.997 (0.990–1.004)
Public facility	2.294 (0.999–5.266)		2.645^*^ (1.120–6.246)
Constant	0.137^*^ (0.0255–0.737)		0.424 (0.0672–2.673)
Observations	2,535		2,535

*** p < 0.001.

** p < 0.01.

* p < 0.05.

Lastly, Table 4 shows that higher Provider Integration Index scores (model 5) were associated with increased odds of clients correctly identifying whether family planning services were available at that facility (OR = 1.153, 95% CI = 1.032–1.288, *p* < 0.05), while Facility Integration Index scores (model 6) did not have a significant effect (OR = 1.043, 95% CI = 0.886–1.227, *p* = 0.614; Table 4). Consistent with results from models 1–4, year was significant while age, education, and desire for another child were insignificant. Family planning client load was significantly associated with correct identification of family planning service availability in both models 5 and 6.

**Table 4:** Association between provider and facility integration index scores and correct identification of family planning service availability at facility.

**Variable**	**OR (95% CI)**		**OR (95% CI)**
**Provider Integration Index Score (Model 5)**	1.153^*^ (1.032–1.288)	**Facility Integration Index Score (Model 6)**	1.043 (0.886–1.227)
Age	1.011 (0.982–1.041)		1.01 (0.982–1.039)
**Education**
None	Ref		Ref
Quranic only	0.642 (0.288–1.429)		0.601 (0.273–1.319)
Primary	1.332 (0.761–2.331)		1.36 (0.779–2.374)
Junior secondary	0.932 (0.509–1.709)		0.936 (0.516–1.698)
Senior secondary	1.553 (0.847–2.850)		1.584 (0.875–2.868)
Higher	1.662 (0.894–3.089)		1.676 (0.903–3.111)
Want another child	1.145 (0.792–1.656)		1.135 (0.779–1.652)
Muslim	0.807 (0.577–1.129)		0.781 (0.561–1.086)
Number of children	1.070 (0.951–1.203)		1.078 (0.958–1.213)
Wealth	1.345^***^ (1.145–1.580)		1.305^**^ (1.111–1.534)
Endline	2.819^***^ (1.863–4.267)		2.976^***^ (1.945–4.554)
**City**
Abuja	Ref		Ref
Benin	1.977 (0.820–4.769)		3.300^**^ (1.489–7.314)
Ibadan	2.392^*^ (1.198–4.777)		3.732^***^ (1.982–7.027)
Ilorin	1.753 (0.912–3.369)		2.245^*^ (1.143–4.408)
Kaduna	1.135 (0.472–2.726)		1.422 (0.586–3.449)
Zaria	2.534 (0.924–6.950)		4.078^**^ (1.592–10.45)
Facility family planning client load	1.014^*^ (1.001–1.028)		1.014^*^ (1.001–1.027)
Public facility	1.558 (0.846–2.871)		1.476 (0.815–2.675)
Constant	0.212 (0.0403–1.112)		0.301 (0.0385–2.364)
Observations	2,535		2,535

*** p < 0.001.

** p < 0.01.

* p < 0.05.

## Discussion

The objective of this study was to utilize Provider and Facility Integration Indexes to identify the relationships between degree of family planning and child immunization services integration and postpartum contraceptive use, receipt of family planning information during immunization visits, and correct identification of family planning services availability among married and cohabitating women attending routine immunization visits. We found that neither Provider nor Facility Integration Index scores were associated with client-reported use of modern contraceptive methods at the time of the clinic visit. We also found that higher Facility Integration Index scores were associated with diminished odds of client-reported receipt of family planning information during an immunization visit; Provider Integration Index scores were not significant. Finally, higher Provider Integration Index scores were associated with increased odds of clients correctly identifying family planning service availability at the facility while Facility Integration Index scores were not. Taken together, our results challenge the assumption that integration provides a well-defined path to improved family planning services delivery and uptake.

The lack of association between the integration index scores and postpartum family planning use indicates that the specific integration approach implemented in these facilities was not sufficient to influence women's contraceptive use. While findings about the effect of integration on contraceptive use are mixed, our result is consistent with prior research, such as that conducted by Nelson (25), that finds family planning and immunization services integration is not associated with increased contraceptive use. Within the facilities in our sample, the lack of association may be due to the integration approach itself or to the degree of fidelity with which the approach was implemented. Numerous studies have highlighted that integration is challenging to implement and contextual factors influence which approach is the most feasible and effective in any given context (24–26, 58). It is also plausible that integration, even if implemented well, plays a limited role in motivating contraceptive use. A substantial body of literature illustrates the profound influence of community factors and social norms on women's contraceptive use. In Nigeria, Alo et al. (59) and Ejembi et al. (60) note that female educational attainment, female autonomy, social support for family planning, and the belief that one's family and friends use family planning are associated with increased contraceptive use while the prevalence of polygyny is associated with decreased use. The characteristics significantly associated with contraceptive use in this study (year, Muslim religion and number of children) reaffirm associations established in the literature (59, 61). Cultural dynamics should be carefully considered when designing an integration approach. Ultimately, our results underscore the need for research that tracks both implementation challenges and fidelity while examining which, if any, specific integration approaches improve contraceptive uptake in various contexts.

The inverse association between Facility Integration Index scores and client-reported receipt of family planning information during immunization visits indicates that maintaining facility management norms that support integrated service delivery may diminish the odds of patients receiving important family planning information. This may be because maintaining these norms places additional workloads on the providers that ultimately compromise information transfer from providers to patients. This would align with findings of studies which show that implementing integrated service delivery may result in less focus allocated to each of the individual services, particularly if one service is considered “secondary” to another (25, 62). Similarly, the lack of association between Provider Integration Index scores and receipt of family planning information during an integrated immunization visit indicates that provider skills and willingness to offer integrated services does not necessarily translate into actual integrated care. This is congruent with the conclusion drawn by Mayhew et al. (48) that integrated physical and human resources infrastructures may not be “sufficient to achieve the integrated delivery of care to clients” as well as the conclusion made by Singer et al. (56) that integrated care may not be delivered even within facilities with the organizational structures and processes in place to do so (48, 56). Providers in our sample frequently missed opportunities to discuss family planning services with women; almost half of the women who did not receive family planning information during immunization services report that they would have been interested in receiving it. This finding aligns with those of Achyut et al. (63) which show that a relatively small proportion of women receive family planning information during integrated maternal health visits.

If women do not receive high-quality family planning information during an integrated visit, they may face diminished ability to confidently choose and use the method that best meets their needs and goals. Women who have attended an integrated consultation of poor quality may opt not to return for a dedicated family planning consultation because they had an unsatisfactory experience, exhausted their available resources, or assumed that they received all the relevant information at the earlier appointment. Our findings raise concerns about the level of, and how best to maintain, family planning service quality within integrated settings. Bolstering integrated services with additional human and financial resources could address constraints, but this may not be feasible in contexts where shortages of trained staff and financial resources are the norm.

Knowledge of family planning service availability is an important first step in accessing these services. It is thus valuable to better understand the role that different dimensions of integration may play in ensuring this awareness. The positive association between Provider Integration Index scores and clients correctly identifying family planning service availability at the facility indicates that having the human resources available to provide family planning services, specifically within integrated child immunization visits, increases client awareness of family planning service availability. This may be because providers at facilities with higher Provider Integration Index scores are more likely to have the skills to provide family planning services at that facility. It may also be because clients attending these facilities have received service availability information through other channels, such as advertisement of family planning service availability or word of mouth from others who have attended the facility and received family planning services. Our findings support the well-established importance of present, skilled, and sufficiently resourced health workers to family planning services provision and awareness raising (44, 64–66).

Our findings and interpretation should be considered in tandem with limitations of this research. Because this study draws upon pooled cross-sectional data, we cannot make causal inferences based upon it. Repeated exposure to family planning messaging during immunization visits or other health facility utilization patterns may be associated with the outcomes of interest; however, data limitations precluded us from ascertaining the number of visits women had made to the interview facility or to other facilities prior to interview. We also do not know whether those who reported using a method had initiated it prior to their initial immunization visit. In this case, their use may not be related to integrated service delivery. Additionally, while we control for desire for more children, we do not control for when women would like to conceive, which would likely influence contraceptive use. Although the vast majority of children are brought to immunization visits by their mothers, it is possible that it could have been another caretaker, which could invalidate our assumption that the interviewed client was in the postpartum period. Finally, this study was conducted in select urban areas of Nigeria in high patient-volume health facilities where NURHI implemented an intervention, which limits generalizability. Notwithstanding these limitations, our study provides valuable insight into health and service delivery outcomes associated with integrated family planning and immunization services.

To our knowledge, this is the first study to analyze family planning and immunization services integration by applying indexes that reflect distinct and uncorrelated dimensions of integration. Applying this approach to various health services can provide a more nuanced understanding of integration. Although integration has the potential to benefit patients, providers, and health systems, our results challenge the idea that integration has a universally positive effect on service delivery, behavioral, and health outcomes. Given the investment and reorganization of resources required to integrate health services, policies should be informed by additional research that identifies appropriate integration models and analyzes whether these models are more efficient and effective than non-integrated models of care.

## Data availability statement

Publicly available datasets were analyzed in this study. Data from this study and all documentation are available upon request through the MLE Dataverse website at: https://dataverse.unc.edu/dataverse/mle.

## Author contributions

KS led the design and implementation of research, data analysis, interpretation of results, and manuscript writing. IS provided critical contextual, theoretical, and methodological guidance and expertise. SC provided theoretical and methodological expertise. MW and JP contributed to conceptualization and presentation of the research. AB contributed to conceptualization and presentation of the research and provided overall guidance and direction. All authors provided critical feedback and helped shape the research, interpretation of results and manuscript, read, and approved the final manuscript.

## Funding

This work was supported, in whole or in part, by the Bill & Melinda Gates Foundation OPP52037. The Foundation provided funding for the data collection of the data used in this study. The Foundation did not play a role in the design of this study or in the analysis or interpretation of data.

## Conflict of interest

Author KS was employed by John Snow, Inc. The remaining authors declare that the research was conducted in the absence of any commercial or financial relationships that could be construed as a potential conflict of interest.

## Publisher's note

All claims expressed in this article are solely those of the authors and do not necessarily represent those of their affiliated organizations, or those of the publisher, the editors and the reviewers. Any product that may be evaluated in this article, or claim that may be made by its manufacturer, is not guaranteed or endorsed by the publisher.

## Figures and Tables

**Figure A1:**
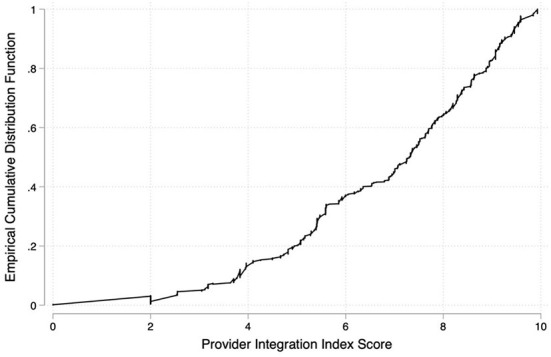
Provider integration index cumulative distribution.

**Figure A2:**
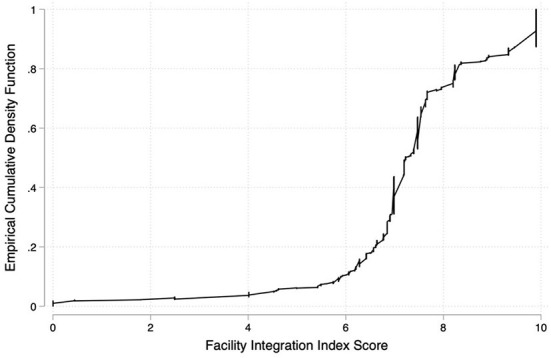
Facility integration index cumulative distribution.

## Appendix

**Table A1:** Provider and facility integration indexes summary statistics.

**Integration index**	**Index score**					
	**Minimum**	**First quartile**	**Median**	**Mean**	**Third quartile**	**Maximum**
**Provider**	**2.00**	**5.29**	**7.30**	**6.80**	**8.57**	**9.93**
**Facility**	**0.00**	**6.85**	**7.23**	**7.30**	**8.20**	**9.90**
